# Cold atmospheric plasma for SARS-CoV-2 inactivation

**DOI:** 10.1063/5.0031332

**Published:** 2020-11-01

**Authors:** Zhitong Chen, Gustavo Garcia, Vaithilingaraja Arumugaswami, Richard E. Wirz

**Affiliations:** 1 Department of Mechanical and Aerospace Engineering, University of California, Los Angeles, California 90095, USA; 2 Department of Molecular and Medical Pharmacology, University of California, Los Angeles, California 90095, USA; 3 Jonsson Comprehensive Cancer Center, UCLA, Los Angeles, California 90095, USA

## Abstract

Syndrome coronavirus 2 (SARS-CoV-2) infectious virions are viable on various surfaces (e.g., plastic, metals, and cardboard) for several hours. This presents a transmission cycle for human infection that can be broken by developing new inactivation approaches. We employed an efficient cold atmospheric plasma (CAP) with argon feed gas to inactivate SARS-CoV-2 on various surfaces including plastic, metal, cardboard, basketball composite leather, football leather, and baseball leather. These results demonstrate the great potential of CAP as a safe and effective means to prevent virus transmission and infections for a wide range of surfaces that experience frequent human contact. Since this is the first-ever demonstration of cold plasma inactivation of SARS-CoV-2, it is a significant milestone in the prevention and treatment of coronavirus disease 2019 (COVID-19) and presents a new opportunity for the scientific, engineering, and medical communities.

Coronavirus disease 2019 (COVID-19) is an emerging infectious disease caused by severe acute respiratory syndrome coronavirus 2 (SARS-CoV-2).^1–3^ Patients with COVID-19 show manifestations of respiratory tract infection, such as fever, cough, pneumonia, and, in severe cases, death. COVID-19 endangers lives and spreads through respiratory droplets, transmitting the virus from one subject to another through air and surface transmission.^4–6^ SARS-CoV-2 has caused a once-in-a-century pandemic, and studies have shown that the infectious virions are viable on various surfaces (e.g., plastic, metals, and cardboard) for several hours.^7–12^ Surface contamination presents a great risk of transmitting SARS-CoV-2 between people, and it is critical to break the transmission cycle by developing new inactivation approaches.

Plasma is one of the four fundamental states of matter (i.e., solid, liquid, gas, and plasma) and was so named since the charged species that comprise the plasma can behave somewhat similar to the cellular components of blood that are bound by blood plasma.^13^ Cold atmospheric plasma (CAP) operating at atmospheric pressure and room temperature has been shown to safely and effectively treat contaminated surfaces and can treat both smooth and highly featured surfaces.^14–16^ The efficacy of CAP is due to its many components, such as reactive oxygen and nitrogen species (RONS), which exhibit favorable behavior for biomedical and industrial applications.^17–24^ In this letter, we employed CAP treatments to inactivate SARS-CoV-2 on various surfaces including plastic, metal, cardboard, basketball composite leather, football leather, and baseball leather.

The CAP was delivered by an atmospheric pressure plasma jet (APPJ) that uses a standard APPJ configuration consisting of a two-electrode assembly with a powered centered electrode connected to a high voltage transformer and a grounded outer ring electrode [Fig. 1(a)], and it is similar to other APPJ designs.^25–29^ The custom body was built using a LulzBot TAZ 6 3D printer at UCLA.^30^ We chose operating conditions that provided relatively stable plasma conditions and a high reactive species content.^31^ These conditions were achieved at an RMS input power of approximately 12 W, and flow rates for argon (Ar) and helium (He) plasmas were 6.4 l/min and 16.5 l/min, respectively. The discharge voltages for both Ar and He feeding gases were 16.8 kV and 16.6 kV (peak–peak) at 12.9 kHz and 12.7 kHz frequency, respectively, as shown in Figs. 1(b) and 1(c). The reactive species content was measured via optical emission spectroscopy (OES) using a fiber-coupled optical spectrometer (LR1-ASEQ Instruments), with a wavelength range of 300 nm–1000 nm. The emission lines and bands of reactive species generated by Ar plasma were identified [Fig. 2(a)].^32^ The high density OH peak was detected at 309 nm, and the low-density O peak was identified at 777 nm. The low-intensity N_2_ second-positive system (*C*^3^Π_u_–*B*^3^Π_g_) with its peaks at 337 nm, 358 nm, and 381 nm was observed. Ar bending was observed in the range of 650 nm and 850 nm.

**FIG. 1.**
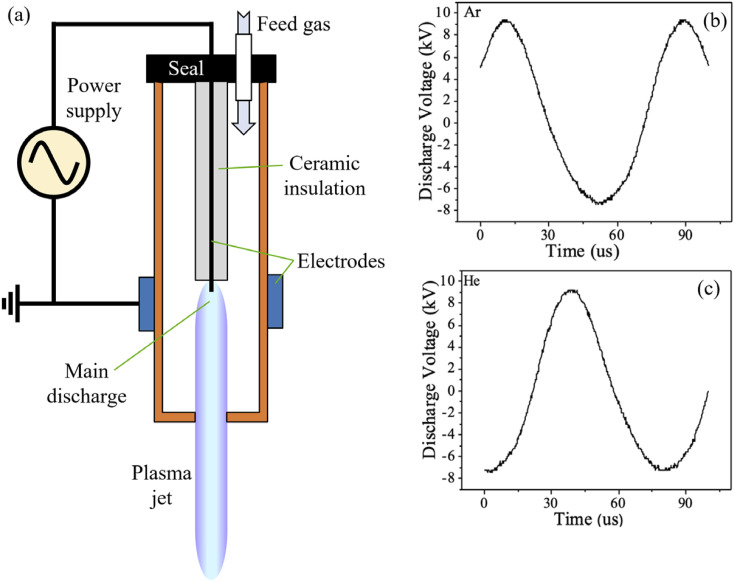
Schematic diagram and discharge voltage traces: (a) a typical schematic diagram of the cold plasma jet device, (b) discharge voltage of the plasma jet using argon as the feeding gas, and (c) discharge voltage of the plasma jet using helium as the feeding gas.

**FIG. 2.**
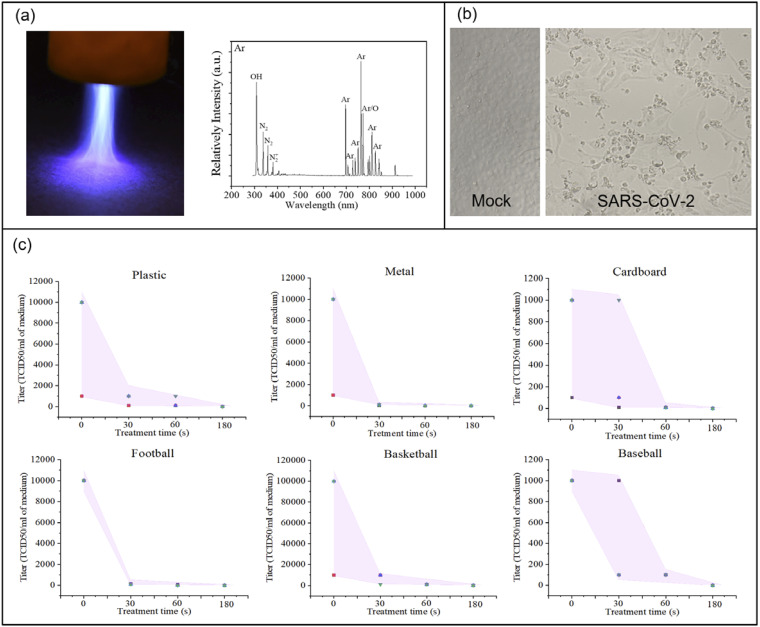
Ar-fed CAP disinfecting SARS-CoV-2: (a) Ar-fed CAP treatment of a plastic surface and the optical emission spectrum of reactive oxygen and nitrogen species (RONS) (exposure: 250 ms), (b) the bright-field image of SARS-CoV-2 infected Vero-E6 cells showing viral cytopathic effect (CPE). Uninfected (mock) cells are included as the control, and (c) SARS-CoV-2 titer response to CAP treatment times of 0 s, 30 s, 60 s, and 180 s on surfaces of plastic, metal, cardboard, leather football, composite leather basketball, and leather baseball. The distance between the plasma device and the surface is ∼15 mm. The error bar in each graph is shown as a shaded area.

The SARS-related coronavirus 2 (SARS-CoV-2) sample, Isolate USA-WA1/2020, was obtained using an oropharyngeal swab from a patient with a respiratory illness who had recently returned from an affected region in China and developed the clinical disease (COVID-19) in January 2020 in Washington, USA. It was received from BEI Resources of the National Institute of Allergy and Infectious Diseases (NIAID). SARS-CoV-2 live culture studies were conducted at UCLA in a BSL-3 high-containment facility. Various surfaces were treated with SARS-CoV-2 at 2 × 10^5^ PFU in 25 *μ*l volume. Virus contaminated surfaces were exposed to helium or argon plasma for various durations. Surfaces contaminated with the virus but not exposed to plasma were included as the control. Infectious SARS-CoV-2 samples were obtained from surfaces by adding 100 *μ*l of DMEM growth media. The titer of the obtained SARS-CoV-2 samples was assessed in Vero-E6 cells in a 96-well format. Each recovered sample was subjected to 10-fold serial dilution, and 100 *μ*l of diluted viral inoculum was added to each well. After 3–4 days postinfection, the wells were examined for the presence of viral cytopathic effect (CPE). At the lowest viral dilution, the wells negative for CPE were identified and included for calculating the virus titer for each sample. SARS-CoV-2 infected Vero-E6 cells show a viral cytopathic effect [Fig. 2(b)].

We observed that Ar-fed CAP treatment inactivated all SARS-CoV-2 on the six surfaces in less than 180 s [Fig. 2(c)] and specifically, metal surfaces exhibited decontamination at 30 s of exposure. Most data points on the plastic and leather football surface showed virus inactivation for 30-s and 60-s treatments. Cardboard and basketball surfaces exhibited effective virus inactivation for a 60-s treatment, while few data points exhibit these effects for 30-s treatments. Additional testing showed similar virus inactivation for cotton cloth material from face masks.

Based on these results, it appears that three important aspects of surface inactivation of SARS-CoV-2 via CAP are material composition, roughness, and absorptivity. When Ar-fed plasma was used to treat the virus on metal surfaces, a higher discharge intensity was observed at the interface. Generally, a higher discharge intensity at the interface will generate more reactive species, and this may explain the highest inactivation efficiency as measured at the metal surface. When compared to the surface roughness of a leather football, a basketball’s surface is much rougher, which appears to lead to a lower inactivation efficiency for the basketball surface. Other surfaces such as the cardboard surface absorb the cultured medium containing SARS-CoV-2, protecting the virus from the discharge and causing lower inactivation efficiency. The baseball surface is a combination of rough and absorbing ones and was measured as having the lowest inactivation efficiency. Based on these results, it can be concluded that surface roughness and absorptivity are dominant factors affecting SARS-CoV-2 inactivation efficiency, while the composition of the surface material is of secondary importance.

Initial testing was performed with helium (He)-fed CAP. Both He-fed and Ar-fed plasmas were operated at atmospheric pressure and room temperature (Fig. 3). To investigate the potential effect of temperature on SARS-CoV-2 inactivation during the exposures,^33^ thermal measurements of the surface were carried out through a tripod-mounted long-wavelength infrared camera (FLIR A655sc) from a distance of approximately 15 cm diagonally above the subject. The center temperature for the Ar plasma-treated surface was ∼32 °C, and that of the He plasma treatment was ∼29 °C. SARS-CoV-2 in solution retained viability for 7 days at a temperature range 20 °C–25 °C and remained viable for 1–2 days at a temperature range 33 °C–37 °C.^34^ Thus, the temperature factor can be neglected here due to the short plasma treatment times used for these tests.

**FIG. 3.**
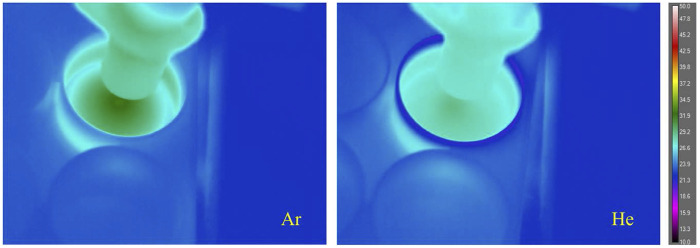
Temperature distribution of Ar and He plasma-treated 6-well plates. The highest temperature for each subject was found at the center of the circular thermal gradient immediately beneath the plasma discharge. The center temperature for the Ar plasma treated surface was ∼32 °C, and for the He plasma treatment, it was ∼29 °C.

Unlike Ar-fed plasma, He-fed plasma did not disinfect all SARS-CoV-2 on metal and plastic surfaces even at 300 s (Fig. 4). This is likely due to the He-fed plasmas having much lower RONS concentrations than Ar-fed plasmas for the same operating conditions [compare Fig. 2(a) for Ar-fed to Fig. 5 for He-fed]. Ar-fed and He-fed plasma jets were detected by the optical emission spectrum at 250 ms and 5000 ms exposure times, respectively. Thus, RONS concentration plays a major role in SARS-CoV-2 inactivation. Future studies will be used to demonstrate higher inactivation rates and shorter treatment times, with target times closer to 10 s–15 s. Additionally, since virus inactivation on organic surfaces was shown, the potential for CAP treatment for clinical applications will also be considered in future studies.

**FIG. 4.**
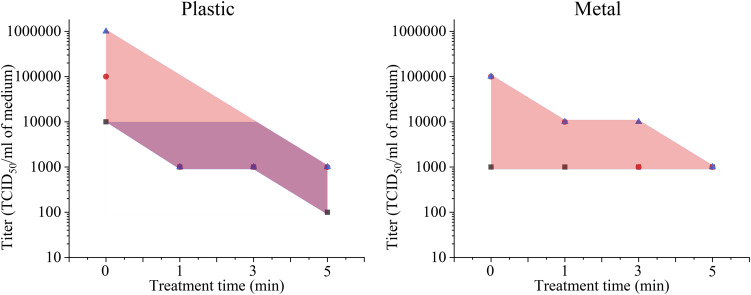
He-fed CAP disinfecting SARS-CoV-2 on plastic and metal surfaces. The distance between the plasma device and the surface is ∼15 mm. The error bar in each graph is shown as a shaded area.

**FIG. 5.**
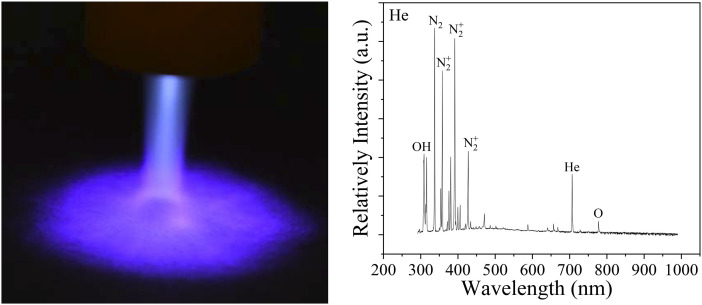
He-fed CAP and its spectrum. Left: the He-fed plasma jet treating surface with plume spreading across the surface. Right: the He-fed plasma jet detected by the optical emission spectrum and containing reactive oxygen and nitrogen species (RONS) (exposure: 5000 ms).

Regarding the possible mechanisms involved in the observed inactivation, SARS-CoV-2 is a positive-sense single-stranded RNA virus and is similar to other coronaviruses to the extent that it is responding to CAP treatments. CAP for inactivation of SARS-CoV-2 should be caused by plasma-generated reactive species inducing virus leakage and functionality loss.^35^ The levels of these reactive species can be adjusted by plasma source design, feeding gas types, operating conditions, the nature of the product/substrate, and the micro-organism itself. Previous studies have highlighted the breakage of structurally important bonds, such as C–C, C–O, and C–N.^36,37^ The charged particle accumulating on the virus’ surface also damages cell membranes through electrostatic disruption.^38^ The electrostatic forces from such an accumulation can exceed the tensile strength of the membrane, leading to its rupture. Reactive species in plasma can induce oxidation of amino acids, nucleic acids, and unsaturated fatty acid peroxides through interaction with membrane lipids, leading to changes in the membranes’ function.^35^ In addition, our recent results show that CAP treatment promotes dendritic cell (DC) maturation in the lymph node, where DCs can present a major histocompatibility complex-peptide to T cells.^30^ The subsequent T cell-mediated immune response can be augmented by immune checkpoint inhibitors, resulting in enhanced local and systemic antiviral immunity. COVID-19-infected pneumonia patients typically have severe immune abnormalities and risk of cytokine release syndrome (CRS), which result into a decrease in T cells and natural killer (NK) cells and an increase in interleukin 6 (IL-6), the CD4/CD8 ratio (CD: cluster of differentiation), fever, tissue/organ dysfunction, and an abnormal coagulation function.^39–41^ Thus, the present results also support the possibility that adoptive therapy with CAP has great potential for COVID-19 immunotherapy.

This letter is the first-ever demonstration of CAP treatment as a viable method for SARS-CoV-2 inactivation. In particular, Ar-fed CAP treatment was shown to quickly and effectively inactivate SARS-CoV-2 on a wide range of surfaces that people frequently touch and therefore has great potential as a safe and effective means to prevent virus transmission and infections. Inactivation efficacy is primarily a factor of surface absorptivity and roughness. Overall, the efficacy of cold plasma for killing SARS-CoV-2 on a variety of surfaces with a wide range of composition, roughness, and absorptivity without damaging the surfaces is encouraging and demonstrates the promising applicability of cold plasma for virus inactivation on surfaces. These results also suggest that cold plasma should be investigated for the inactivation of aerosol-borne SARS-CoV-2. Since cold plasma is significantly safer than most other treatment methods such as alcohol, UV radiation, and the like, this work opens a wide range of opportunities for the scientific, engineering, and medical communities.

## Data Availability

The data that support the findings of this study are available from the corresponding author upon request.
